# Echocardiographic characteristics of patients with acute heart failure requiring tolvaptan: a retrospective study

**DOI:** 10.1186/s12947-015-0022-7

**Published:** 2015-06-08

**Authors:** Yasuki Nakada, Satoshi Okayama, Tomoya Nakano, Tomoya Ueda, Kenji Onoue, Yukiji Takeda, Rika Kawakami, Manabu Horii, Shiro Uemura, Shinichi Fujimoto, Yoshihiko Saito

**Affiliations:** First Department of Internal Medicine, Nara Medical University, 840 Shijo-cho, Kashihara Nara, 634-8522 Japan; Department of Cardiovascular Internal Medicine, Nara City Hospital, Nara, Japan; Education Development Center, Nara Medical University, Nara, Japan

**Keywords:** Diuretics, Cardiorenal syndrome, Renal dysfunction, Renal failure, Tolvaptan, Left atrium, Inferior vena cava, Tricuspid regurgitation

## Abstract

**Background:**

No study has investigated the admission echocardiographic characteristics of acute heart failure (AHF) patients who are resistant to conventional diuretics and require tolvaptan.

**Methods:**

We retrospectively analyzed the echocardiographic characteristics of AHF patients who were resistant to conventional diuretics and took tolvaptan (tolvaptan group: 26 patients), and compared them to those who were sensitive to conventional diuretics (conventional group: 180 patients).

**Results:**

The tolvaptan group had a higher left atrial volume index (96.0 ± 85.0 mL/m2 vs. 45.8 ± 25.9 mL/m^2^, *p* < 0.0001), maximum inferior vena cava diameter (20.7 ± 6.9 mm vs. 18.1 ± 4.2 mm, *p* < 0.01), and higher tricuspid regurgitation grade (1.1 ± 0.8 vs. 0.8 ± 0.6, *p* < 0.05) than the conventional group. However, the left ventricular ejection fraction and end diastolic diameter were similar between the groups. Responders of tolvaptan had no significant echocardiographic differences compared to the non-responders.

**Conclusions:**

The admission echocardiographic characteristics of AHF patients requiring tolvaptan included a larger left atrium, inferior vena cava, and more severe tricuspid regurgitation. Echocardiography may provide useful information for the early and appropriate initiation of tolvaptan.

## Background

The regulation of cardiac volume and pressure overload is one of the most important therapeutic strategies for acute heart failure (AHF) [1, 2]. Cardiac volume overload can be ameliorated by the appropriate restriction of sodium and water intake and conventional diuretics such as loop diuretics in many patients with AHF; however, it is difficult to be ameliorated in some patients, because conventional diuretics are less effective or cannot be administered in sufficient doses due to worsening renal function and electrolyte imbalances [3].

Tolvaptan (Otsuka Pharmaceutical Co., Ltd., Tokyo, Japan) is an oral selective vasopressin type 2 receptor antagonist that promotes water excretion without changing the renal sodium and potassium excretion [4]. Large clinical studies have shown that tolvaptan can rapidly improve the congestive symptoms of AHF and reduce body weight without serious adverse events [5, 6]. Tolvaptan improves the long-term prognosis, especially in AHF patients with severe hyponatremia [7]; moreover, even with normonatremia, the selection of low-dose tolvaptan can improve congestive symptoms without causing hypernatremia [8]. Tolvaptan is expected to be widely used in patients with AHF.

Yet, the following two problems remain unresolved. First, the decision to initiate tolvaptan is difficult immediately after admission. In Japan, tolvaptan is indicated for the treatment of volume overload in AHF patients who are resistant to conventional diuretics, and it should not be administered in all AHF patients because of the heavy economic burden to patients and society. However, tolvaptan is difficult to administer to only patients who are accurately identified as being resistant to conventional diuretics immediately after admission. Even if resistance is suspected, an increase in the dose will be attempted in many patients [9], without initiating tolvaptan. We consider that some patients with AHF cannot possibly obtain sufficient clinical benefits from tolvaptan because of a delayed decision. Second, the early prediction of responsiveness to tolvaptan is difficult.

The purposes of this study were (1) to determine the admission echocardiographic characteristics of AHF patients who are resistant to conventional diuretics and thus require tolvaptan by comparing them to those who are sensitive to conventional diuretics; and (2) to investigate the admission echocardiographic differences between responders and non-responders of tolvaptan. We consider that these echocardiographic indices will be useful for the early and appropriate initiation of tolvaptan.

## Methods

### Study patients

From April 2011 to December 2013, there were 328 patients hospitalized in our department who were diagnosed with AHF according to the Framingham criteria, and they were prospectively registered in our database [10, 11]. We collected the clinical data of AHF patients who were resistant to conventional diuretic therapy and thus took tolvaptan orally (tolvaptan group) and that of those who were sensitive to conventional diuretic therapy (conventional group). Tolvaptan treatment was initiated by the attending doctors’ decision, which was in accordance with the health insurance treatment of Japan. Conventional diuretic therapy was defined as the combination of diuretics excluding tolvaptan, beta-blockers, renin-angiotensin system inhibitors (e.g., angiotensin-converting enzyme inhibitors or angiotensin II receptor blockers), vasodilators, or inotropic agents (e.g., digoxin, dobutamine, and phosphodiesterase-III inhibitors). The exclusion criteria were as follows: (1) transthoracic echocardiography was not performed within 48 h after admission in the central physiological laboratory; (2) the echocardiography had poor image quality and insufficient data; (3) coronary revascularization, such as coronary artery bypass grafting and percutaneous coronary intervention, and devise therapy, such as pacemakers, implantable cardioverter-defibrillators, and cardiac resynchronization therapy were considered to be significantly effective for improving the congestive symptoms of AHF or these were performed during hospitalization; and (4) patients with acute coronary syndrome, acute myocarditis, or acute pulmonary embolism. Thus, we analyzed 206 patients with AHF. The present study was approved by the Nara Medical University Institutional Ethics Committee and was performed under the clinical research protocols in accordance with the 1975 Declaration of Helsinki.

### Clinical characteristics

The following clinical data were collected on admission: age, sex, body mass index (BMI), etiology of heart failure, presence of hypertension, diabetes mellitus, dyslipidemia, heart rate, and blood pressure and laboratory data. Hypertension was defined as the current use of an anti-hypertensive drug for the treatment of blood pressure (BP) elevation (systolic BP, ≥140 mmHg; diastolic BP, ≥90 mmHg). Diabetes mellitus was defined as undergoing current treatment with hypoglycemic agents, a non-fasting blood glucose level of ≥200 mg/dL, or a fasting blood glucose level of ≥126 mg/dL. Dyslipidemia was defined as the current use of a statin or fibrate, a low-density lipoprotein cholesterol level of ≥140 mg/dL, a high-density lipoprotein cholesterol level of <40 mg/dL, or a triglyceride level of ≥150 mg/dL. The estimated glomerular filtration rate (eGFR) was calculated according to the published equation for Japanese persons: 194 × serum creatinine ^−1.094^ × age^−0.287^ × (0.739 for females) [12].

### Echocardiography

Ultrasound examinations were performed using the Sonos 7500 systems (Philips, Best, the Netherlands) and Acuson Sequoia systems (Siemens, Erlangen, Germany). Left ventricular ejection fraction (LVEF) and end-diastolic volume (LVEDV) were calculated by using the modified Simpson’s method. The left atrial (LA) volume was calculated using the biplane area-length technique. The LV end-diastolic diameter (LVDd), LV end-systolic diameter (LVDs), septal wall thickness (SWT), posterior wall thickness (PWT), and LA diameter were measured using M-mode echocardiography [13]. Diastolic function was assessed using transmitral and tissue Doppler imaging at the septal mitral annulus. Peak early (E) and late transmitral filling velocities (A), their ratio, the deceleration time of peak E velocity, early diastolic mitral annular velocity (e’), and E/e’ were measured. Subjects were classified into the following four groups according to the Recommendations for the Evaluation of Left Ventricular Diastolic Function by the American Society of Echocardiography: normal, mild, moderate, and severe [14]. The severity of valvular regurgitation was quantitatively evaluated with Doppler color flow imaging, in which the vena contracta width was measured at the narrowest portion of the regurgitant jet [15]. Aortic and mitral regurgitation was graded on a 4-point scale: none or trivial (score 0), mild (score 1), moderate (score 2), or severe (score 3). Tricuspid regurgitation was graded on a 3-point scale: none or trivial (score 0), mild or moderate (score 1), or severe (score 2). The inferior vena cava (IVC) diameter was measured, and the collapsibility index was calculated as a percentage of the difference between the maximal and minimal diameter [16].

### Definitions

We defined patients who responded to tolvaptan (responders) as those with a ≥50 % increase in the maximum 24-h urine volume within 3 days after administration compared to before administration. Non-responders were those with a <50 % increase according to the report by Sakaguchi et al. [17].

### Statistical analysis

Data are presented as a mean ± standard deviation. The chi-square or Fisher’s exact tests were used to analyze univariate associations between the categorical variables, and the Mann–Whitney *U* test was used for the continuous variables. We used JMP software (SAS Institute, Cary, NC, USA) to analyze the data. *P* values <0.05 were considered statistically significant.

## Results

### Clinical characteristics

**Fig. 1:**
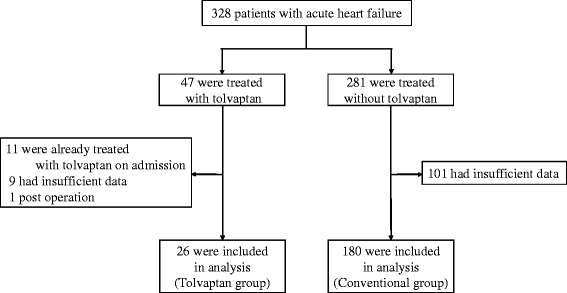
Enrollment

The tolvaptan and conventional groups consisted of 26 and 180 AHF patients, respectively (Fig. 1). The tolvaptan group included 16 patients who were admitted for scheduled initiation because of ≥2 prior hospitalizations, 8 who were treated by tolvaptan after ≥3 days of conventional therapy, and 2 who had severe weight gain (7 kg and 16 kg) due to fluid retention.

Tolvaptan was administered at a mean initial dose of 8.2 ± 3.7 mg/day. The clinical characteristics on admission are shown in Table 1. There were no significant differences in age, sex, BMI, etiology of heart failure, or comorbid diseases excluding atrial fibrillation between the groups. Previous AHF hospitalization (2.1 ± 1.7 times vs. 0.5 ± 0.9 times, *p* < 0.001) and atrial fibrillation (73 % vs. 38 %, *p* < 0.001) were significantly more common in the tolvaptan group than the conventional group. The tolvaptan group had a significantly lower heart rate (79 ± 18 bpm vs. 96 ± 27 bpm, *p* < 0.001), systolic blood pressure (123 ± 27 mmHg vs. 150 ± 38 mmHg, *p* < 0.001), diastolic blood pressure (71 ± 17 mmHg vs. 84 ± 22 mmHg, *p* < 0.01), and hemoglobin (10.3 ± 2.1 g/dL vs. 11.5 ± 2.3 g/dL, *p* < 0.05). The eGFR was significantly lower (30.7 ± 17.6 mL/min/1.73 m^2^ vs. 50.1 ± 27.3 mL/min/1.73 m^2^, *p* < 0.001) and severe renal dysfunction (eGFR <30 mL/min/1.73 m^2^) was more commonly observed in the tolvaptan group than in the conventional group (58 % vs. 26 %, *p* < 0.005). There were no differences in the concentration of sodium, potassium, and B-type natriuretic peptide (BNP) between the groups. The administration of β-blockers (69 % vs. 33 %, *p* < 0.001), renin-angiotensin system inhibitors (77 % vs. 54 %, *p* < 0.05), loop diuretics (100 % vs. 51 %, *p* < 0.001), thiazide diuretics (26 % vs. 9 %, *p* < 0.005), and aldosterone blockers (54 % vs. 18 %, *p* < 0.05) was significantly more common in the tolvaptan group than in the conventional group.

**Table 1:** Clinical characteristics

	Tolvaptan group	Conventional group	*p* value
Number	26	180	
Age (years)	75 ± 11	74 ± 12	0.88
Sex (male,%)	15 (58)	96 (53)	0.68
BMI (kg/m^2^)	23.8 ± 4.4	23.6 ± 4.4	0.77
Previous HF hospitalization	2.1 ± 1.7	0.5 ± 0.9	<0.001
Etiology of heart failure			
Ischemic heart disease	8 (31)	68 (38)	0.87
Dilated cardiomyopathy	5 (19)	30 (17)	
Valvular heart disease	5 (19)	26 (14)	
Others	8 (31)	56 (31)	
Comorbid disease			
Hypertension	20 (77)	136 (76)	0.88
Diabetes mellitus	14 (54)	81 (45)	0.40
Dyslipidemia	10 (38)	73 (41)	0.84
Atrial fibrillation	19 (73)	69 (38)	<0.001
Vital sign on admission			
Heart rate (bpm)	79 ± 18	96 ± 27	<0.001
SBP (mmHg)	123 ± 27	150 ± 38	<0.001
DBP (mmHg)	71 ± 17	84 ± 22	<0.01
Blood chemistry on admission			
Hemoglobin (g/dl)	10.3 ± 2.1	11.5 ± 2.3	<0.05
BUN (mg/dl)	48 ± 27	28 ± 17	<0.001
Scr (mg/dl)	2.0 ± 1.0	1.5 ± 1.4	<0.001
eGFR (ml/min/1.73 m^2^)	30.7 ± 17.6	50.1 ± 27.3	<0.001
eGFR <30 ml/min/1.73 m^2^ (%)	15 (58)	47 (26)	<0.005
Na (mEq/l)	137 ± 6	139 ± 4	0.22
K (mEq/l)	4.3 ± 1.0	4.2 ± 0.7	0.38
BNP (pg/ml)	746 (241–1527)	845 (465–1693)	0.23
Medication on admission			
β-blockers	18 (69)	59 (33)	<0.001
RAS inhibitors	20 (77)	98 (54)	<0.05
Loop diuretics	26 (100)	92 (51)	<0.001
Thiazide diuretics	8 (26)	16 (9)	<0.005
Aldosterone antagonists	14 (54)	32 (18)	<0.05
Device therapy	7 (27)	28 (16)	0.15

The data are presented as the mean ± SD for continuous normally distributed variables, the median (25th to 75th interquartile range [IQR]) for continuous non-normally distributed variables, or n (%). *BMI* body mass index, *SBP* systolic blood pressure. *DBP* diastolic blood pressure. *BUN* blood urea nitrogen, *Scr* serum creatinine, *eGFR* estimated glomerular filtration rate, *BNP* brain natriuretic peptide, *RAS* renin-angiotensin system

### Echocardiography

The echocardiographic parameters are summarized in Table 2. There were no significant differences in the LVEF, LVEDV index, LVDd, LVDs, SWT, and PWT between the groups. However, the LA diameter (53.4 ± 9.9 mm vs. 47.1 ± 8.5 mm, *p* < 0.001) and volume index (96.0 ± 85.0 mL/m^2^ vs. 45.8 ± 25.9 mL/m^2^, *p* < 0.0001) were significantly larger in the tolvaptan group than in the conventional group. In the tolvaptan group, the maximum IVC diameter was significantly larger (20.7 ± 6.9 mm vs. 18.1 ± 4.2 mm, *p* < 0.01), and the collapsibility index was significantly lower (0.35 ± 0.16 vs. 0.46 ± 0.15, *p* < 0.001). While there was no significant difference in the severity score and vena contracta width of aortic and mitral regurgitation between the groups, tricuspid regurgitation was significantly more severe in the tolvaptan group than in the conventional group (severity score: 1.1 ± 0.8 vs. 0.8 ± 0.6, *p* < 0.05; vena contracta width: 5.8 ± 5.1 mm vs. 3.6 ± 3.2 mm, *p* < 0.005). Evaluating diastolic function was difficult in patients with atrial fibrillation; thus, it could only be evaluated in 7 patients of the tolvaptan group and in 111 patients of the conventional group. The tolvaptan group tended to have more severe diastolic dysfunction than the conventional group. The tricuspid regurgitation pressure gradient (TRPG) was measured in all patients of the tolvaptan group; however, it could only be measured in 162 patients of the conventional group with TR. The TRPG was similar between the groups (40.7 ± 13.0 mmHg vs. 42.3 ± 16.2 mmHg, *p* = 0.63).

**Table 2:** Echocardiographic data

	Tolvaptan group	Conventional group	*p* value
Number	26	180	
2 dimensional echocardiography			
LVEF (%)	45.8 ± 17.7	45.3 ± 17.0	0.84
LVDd (mm)	53.8 ± 10.1	55.4 ± 9.8	0.35
LVDs (mm)	41.4 ± 11.9	42.4 ± 12.0	0.67
LV end-diastolic volume index	93.5 ± 39.2	102.4 ± 38.1	0.21
SWT (mm)	9.8 ± 2.5	10.6 ± 2.9	0.15
PWT (mm)	10.0 ± 2.0	10.4 ± 2.1	0.34
LA diameter (mm)	53.4 ± 9.9	47.1 ± 8.5	<0.001
LA volume index (ml/m2)	96.0 ± 85.0	45.8 ± 25.9	<0.0001
Maximum IVC diameter (mm)	20.7 ± 6.9	18.1 ± 4.2	<0.01
IVC collapsibility index	0.35 ± 0.16	0.46 ± 0.15	<0.001
Doppler echocardiography			
Diastolic dysfunction grade			
Mild	0	19	0.06
Moderate	1	27	
Severe	6	38	
E/A	2.1 ± 0.4	1.4 ± 0.8	<0.005
DT (ms)	173 ± 43	178 ± 55	0.23
E/e’	25.2 ± 19.5	22.4 ± 11.5	0.83
Valvular regurgitation			
AR severity grade	0.7 ± 1.1	0.7 ± 1.0	0.88
AR jet vena contracta (mm)	1.5 ± 2.4	1.5 ± 2.5	0.88
MR severity grade	1.7 ± 0.8	1.6 ± 1.0	0.47
MR jet vena contracta (mm)	4.5 ± 2.6	4.1 ± 2.8	0.57
TR severity grade	1.1 ± 0.8	0.8 ± 0.6	<0.05
TR jet vena contracta (mm)	5.8 ± 5.1	3.6 ± 3.2	<0.005
TR pressure gradient (mmHg)	40.7 ± 13.0	42.3 ± 16.2	0.63

*LVEF* left ventricular ejection fraction, *LVDd* left ventricular end-diastolic dimension, *LVDs* left ventricular end-systolic dimension, *LVEDVi* left ventricular end-diastolic volume index, *SWT* septal wall thickness, *PWT* posterior wall thickness, *LA* left atrium, *AR* aortic regurgitation, *MR* mitral regurgitation, *TR* tricuspid regurgitation, *max IVC* max inferior vena cava, *E/A* early/atrial transmitral flow velocity, *DT* deceleration time, *E/e’* early transmitral flow velocity/early mitral annular velocity

### Prediction of responsiveness to tolvaptan

In the tolvaptan group, there were 13 responders and 13 non-responders. Their clinical and echocardiographic characteristics on admission are shown in Tables 3, 4 and 5. BNP tended to be lower in the responders than in the non-responders (475 pg/mL vs. 917 pg/mL, *p* = 0.07). In limited patients who had no renin-angiotensin system inhibitors, the diastolic blood pressure was significantly higher in the responders than in the non-responders (77 ± 9 mmHg vs. 54 ± 5 mmHg, *p* < 0.05). There were no significant echocardiographic differences between the groups.

**Table 3:** Clinical characteristics in responder and non-responder

	Responder	Non-responder	p value
Number	13	13	
Initial dose (mg)	8.9 ± 4.5	7.5 ± 2.7	0.56
Age (years)	74.0 ± 12.0	76.0 ± 10.0	0.76
Sex (male, %)	9 (69)	6 (46)	0.23
BMI (kg/m^2^)	23.5 ± 3.8	24.1 ± 5.1	0.64
Previous HF hospitalization	2.5	1.7	0.35
Etiology of heart failure			
Ischemic heart disease	3 (23)	5 (38)	0.70
Dilated cardiomyopathy	2 (15)	3 (23)	
Valvular heart disease	3 (23)	2 (15)	
Others	5 (38)	3 (23)	
Comorbid disease			
Hypertension	9 (69)	11 (85)	0.35
Diabetes mellitus	6 (46)	8 (62)	0.43
Dyslipidemia	3 (23)	7 (54)	0.10
Atrial fibrillation	10 (69)	9 (77)	0.69
Vital sign on admission			
Heart rate (bpm)	77 ± 11	80 ± 24	0.74
SBP (mmHg)	117 ± 23	129 ± 29	0.28
DBP (mmHg)	71 ± 15	72 ± 19	0.76
Blood chemistry on admission			
Hemoglobin (g/dl)	10.6 ± 2.5	10.0 ± 1.7	0.72
BUN (mg/dl)	42.0 ± 26.0	54.0 ± 29.0	0.23
Scr (mg/dl)	1.9 ± 1.0	2.1 ± 1.0	0.59
eGFR (ml/min/1.73 m^2^)	32.8 ± 17.3	28.6 ± 18.3	0.54
Na (mEq/l)	136.0 ± 6.0	138.0 ± 5.0	0.34
K (mEq/l)	4.3 ± 1.2	4.3 ± 0.6	0.37
BNP (pg/ml)	475 (151–1106)	917 (484–1766)	0.07
Medication on admission			
β-blockers	8 (62)	10 (77)	0.42
RAS inhibitors	10 (77)	10 (77)	1.00
Loop diuretics	13 (100)	13 (100)	1.00
Thiazide diuretics	6 (46)	2 (15)	0.10
Aldosterone antagonists	8 (62)	6 (46)	0.46
Device therapy	3 (23)	4 (31)	0.69

Abbreviations are defined in Table 1

**Table 4:** Effects of antihypertensive agents on heart rate and blood pressure in responder and non-responder

	Responder	Non-responder	*p* value
Administration of β blockers			
Number	8	10	
Heart rate (bpm)	77 ± 11	80 ± 18	0.72
SBP (mmHg)	108 ± 24	128 ± 30	0.14
DBP (mmHg)	62 ± 11	72 ± 22	0.26
No β blockers			
Number	5	3	
Heart rate (bpm)	77 ± 12	82 ± 45	0.81
SBP (mmHg)	132 ± 11	130 ± 33	0.91
DBP (mmHg)	84 ± 12	69 ± 8	0.09
Administration of RAS inhibitors			
Number	10	10	
Heart rate (bpm)	78 ± 12	84 ± 27	0.58
SBP (mmHg)	118 ± 24	136 ± 29	0.17
DBP (mmHg)	69 ± 17	77 ± 19	0.32
No RAS inhibitors			
Number	3	3	
Heart rate (bpm)	74 ± 2	70 ± 5	0.29
SBP (mmHg)	113 ± 23	105 ± 17	0.66
DBP (mmHg)	77 ± 9	54 ± 5	<0.05

Abbreviations are defined in Table 1

**Table 5:** Echocardiographic data in responder and non-responder

	Responder	Non-responder	p value
Number	13	13	
2 dimensional echocardiography			
LVEF (%)	42.5 ± 19.0	49.1 ± 16.3	0.37
LVDd (mm)	52.4 ± 10.4	55.2 ± 9.9	0.40
LVDs (mm)	41.3 ± 12.3	41.5 ± 12.0	0.94
LVEDVi	86.5 ± 40.2	100.6 ± 38.6	0.26
SWT (mm)	9.3 ± 2.8	10.2 ± 2.1	0.28
PWT (mm)	10.0 ± 2.5	10.1 ± 1.5	0.75
LAD (mm)	53.4 ± 10.7	53.3 ± 9.5	0.98
LA volume index (ml/m2)	111.9 ± 115.9	84.4 ± 57.1	0.50
Maximum IVC diameter (mm)	21.8 ± 9.1	19.6 ± 4.1	0.43
IVC collapsibility index	0.34 ± 0.15	0.4 ± 0.2	0.80
Doppler echocardiography			
Valvular regurgitation			
AR severity grade	0.5 ± 1.1	0.8 ± 1.1	0.59
AR jet vena contracta (mm)	1.1 ± 2.1	1.8 ± 2.6	0.40
MR severity grade	1.7 ± 0.8	1.8 ± 0.9	0.82
MR jet vena contracta (mm)	4.0 ± 2.2	4.8 ± 2.9	0.48
TR severity grade	1.2 ± 0.8	1.1 ± 0.8	0.80
TR jet vena contracta (mm)	6.6 ± 6.6	5.1 ± 3.5	0.48
TR pressure gradient (mmHg)	38.6 ± 15.0	42.7 ± 11.0	0.44

Abbreviations are defined in Table 2

## Discussion

This is the first study to report on the admission echocardiographic characteristics of AHF patients who are resistant to conventional diuretics and thus require tolvaptan, and we compared these patients with those who are sensitive to conventional diuretics. The characteristics included a larger LA and IVC and more severe TR.

The reasons why AHF patients requiring tolvaptan had these echocardiographic characteristics can not be sufficiently clarified in this study. However, on the basis of several studies, we speculate that AHF patients with these characteristics frequently have elevated central and renal venous pressure, which worsens their renal function. Renal dysfunction can reduce responsiveness to loop diuretics through impaired tubular delivery due to diminished renal blood flow and reduced activity in the proximal tubular carrier system caused by competition from the accumulated organic anions [3].

The LA size reflects the LV diastolic function [18, 19]. Diastolic dysfunction can induce pulmonary hypertension through the passive transmission of elevated end diastolic pressures, reactive pulmonary vasoconstriction, and vascular remodeling [20]. Pulmonary hypertension can induce functional tricuspid regurgitation through annular dilatation and tethering of the leaflets due to right ventricular dilatation and dysfunction [21], which elevates the central and renal venous pressure. The elevated central venous pressure manifests as IVC dilatation [22].

Renal congestion as well as low cardiac output is closely related to the progression of renal dysfunction. Many clinical studies have shown that patients with heart failure and renal congestion commonly have worsening renal function independent of cardiac output [23–27]. Tanaka et al. demonstrated that renal congestion contributed to worsening renal function by augmenting oxidative stress-mediated inflammation in the tubulointerstitium in a histopathological study on patients with non-ischemic heart failure and renal dysfunction [24]. A few basic studies have been reported on this relationship between renal congestion and dysfunction [28, 29]. Doty et al. demonstrated that elevating the renal venous pressure by 30 mmHg for 2 h can decrease the renal artery blood flow and glomerular filtration rate while increasing the plasma renin activity, serum aldosterone, and urinary protein leak in experimental swine models with stable cardiac index and arterial pressure [28]. To verify this, further investigations that include a combination of echocardiography and invasive hemodynamic monitoring are required.

Recently, the earlier initiation of tolvaptan has been shown to prevent the exacerbation of acute kidney injury and improve the prognosis of patients with AHF [30]. Moreover, some basic studies have shown that tolvaptan has cardio- and renal-protective effects [31–35], whereas the volume reduction by loop diuretics leads to a decrease in the renal blood flow and activation of the renin-angiotensin-aldosterone and sympathetic nervous systems [36]. Morooka et al. demonstrated that tolvaptan can ameliorate glomerulosclerosis and tubulointerstitial fibrosis and improve renal dysfunction concomitantly by suppressing activated V2R, V1aR, renin, endothelin-1, aquaporin-2 in rats with hypertensive heart failure [32]. According to our clinical and echocardiographic data, patients who will obtain great benefit from tolvaptan therapy may present the following profiles in addition to the resistance to conventional diuretics: 1) left atrial dilatation, 2) severe tricuspid valve regurgitation, 3) IVC dilatation, 4) atrial fibrillation, and 5) renal dysfunction. By accurately assessing these profiles, tolvaptan can be initiated as early as possible in appropriate AHF patients, and this is considered important for achieving the maximum effects.

Non-responders to tolvaptan are observed in 18–39 % of patients with AHF [37–39], and establishing a prediction method for responsiveness to tolvaptan is important for its effective use. Our study detected no significant differences between the responders and non-responders to tolvaptan, and this was probably because of the small number of subjects. However, the following prediction methods have been reported. First, a baseline urine osmolality of >352 mOsm/L that decreases >26 % between 4–6 h after administration has been noted in responders to tolvaptan [37]. Second, according to echocardiographic evaluation, the tricuspid annular plane systolic excursion and right ventricular wall strain are impaired in non-responders to tolvaptan [38]. Third, the serum concentration of blood urea nitrogen 1 day after tolvaptan initiation is significantly increased in non-responders to tolvaptan [39].

### Study limitations

The present study has the following limitations. First, we retrospectively analyzed the data from a small number of subjects at a single center. Second, resistance to conventional diuretics was not defined, and a detailed standard for initiating tolvaptan was not determined. However, all patients in the tolvaptan group had other diuretics, ≥2 prior hospitalizations, ≥3 days of conventional therapy, or severe weight gain due to fluid retention. Moreover, since patients who could be treated by coronary revascularization or devise therapy were excluded from this study, we considered that they were resistant to conventional diuretics and thus required tolvaptan. Third, the diastolic function and TRPG were similar between the tolvaptan and conventional group, which may be because these indicators could not be measured in some patients. Moreover, TRPG may be poorly correlated to the severity of TR, because the modified Bernoulli equation is not exactly satisfied in patients with severe tricuspid valve regurgitation and dehiscence due to dilated annulus. Further investigations are required to overcome these limitations.

In conclusion, patients with AHF who were resistant to conventional diuretics and thus required tolvaptan had a larger LA and IVC and more severe tricuspid regurgitation according to their admission echocardiography. Therefore, echocardiography on admission may provide useful information for deciding when to initiate tolvaptan in patients with AHF.

## Abbreviations

AHF: Acute heart failure
BMI: Body mass index
BP: Blood pressure
eGFR: Estimated glomerular filtration rate
LVEF: Left ventricular ejection fraction
LVEDV: end-diastolic volume
LA: Left atrium
LVDd: LV end-diastolic diameter
LVDs: LV end-systolic diameter
SWT: Septal wall thickness
PWT: Posterior wall thickness
E: Peak early transmitral filling velocity
A: Late transmitral filling velocity
e’: Early diastolic mitral annular velocity
IVC: Inferior vena cava
TRPG: Tricuspid regurgitation pressure gradient

## References

[1] Dickstein K, Cohen-Solal A, Filippatos G, McMurray JJ, Ponikowski P, Poole-Wilson PA (2008). ESC guidelines for the diagnosis and treatment of acute and chronic heart failure 2008: the task force for the diagnosis and treatment of acute and chronic heart failure 2008 of the European society of cardiology. Developed in collaboration with the heart failure association of the ESC (HFA) and endorsed by the European society of intensive care medicine (ESICM). Eur J Heart Fail.

[2] Levy PD, Bellou A. Acute heart failure treatment. Curr Emerg Hosp Med Rep. 2013;1 10.1007/s40138-013-0012-8 PMC3821770 24223323

[3] Krämer BK, Schweda F, Riegger GA (1999). Diuretic treatment and diuretic resistance in heart failure. Am J Med.

[4] Costello-Boerrigter LC, Smith WB, Boerrigter G, Ouyang J, Zimmer CA, Orlandi C (2006). Vasopressin-2-receptor antagonism augments water excretion without changes in renal hemodynamics or sodium and potassium excretion in human heart failure. Am J Physiol Renal Physiol.

[5] Gheorghiade M, Konstam MA, Burnett JC, Grinfeld L, Maggioni AP, Swedberg K (2007). Short-term clinical effects of tolvaptan, an oral vasopressin antagonist, in patients hospitalized for heart failure: the EVEREST clinical status trials. JAMA.

[6] Konstam MA, Gheorghiade M, Burnett JC, Grinfeld L, Maggioni AP, Swedberg K (2007). Effects of oral tolvaptan in patients hospitalized for worsening heart failure: the EVEREST Outcome Trial. JAMA.

[7] Hauptman PJ, Burnett J, Gheorghiade M, Grinfeld L, Konstam MA, Kostic D (2013). Clinical course of patients with hyponatremia and decompensated systolic heart failure and the effect of vasopressin receptor antagonism with tolvaptan. J Card Fail.

[8] Kinugawa K, Sato N, Inomata T, Shimakawa T, Iwatake N, Mizuguchi K (2014). Efficacy and safety of tolvaptan in heart failure patients with volume overload. Circ J.

[9] Cox ZL, Lenihan DJ (2014). Loop diuretic resistance in heart failure: resistance etiology-based strategies to restoring diuretic efficacy. J Card Fail.

[10] Ueda T, Kawakami R, Horii M, Sugawara Y, Matsumoto T, Okada S (2013). High mean corpuscular volume is a new indicator of prognosis in acute decompensated heart failure. Circ J.

[11] Ueda T, Kawakami R, Horii M, Sugawara Y, Matsumoto T, Okada S (2014). Noncardiovascular death, especially infection, is a significant cause of death in elderly patients with acutely decompensated heart failure. J Card Fail.

[12] Matsuo S, Imai E, Horio M, Yasuda Y, Tomita K, Nitta K (2009). Revised equations for estimated GFR from serum creatinine in Japan. Am J Kidney Dis.

[13] Lang RM, Badano LP, Mor-Avi V, Afilalo J, Armstrong A, Ernande L (2015). Recommendations for cardiac chamber quantification by echocardiography in adults: an update from the American society of echocardiography and the European association of cardiovascular imaging. J Am Soc Echocardiogr.

[14] Nagueh SF, Appleton CP, Gillebert TC, Marino PN, Oh JK, Smiseth OA (2009). Recommendations for the evaluation of left ventricular diastolic function by echocardiography. Eur J Echocardiogr.

[15] Lancellotti P, Tribouilloy C, Hagendorff A, Popescu BA, Edvardsen T, Pierard LA (2013). Recommendations for the echocardiographic assessment of native valvular regurgitation: an executive summary from the European association of cardiovascular imaging. Eur Heart J Cardiovasc Imaging.

[16] Moreno FL, Hagan AD, Holmen JR, Pryor TA, Strickland RD, Castle CH (1984). Evaluation of size and dynamics of the inferior vena cava as an index of right-sided cardiac function. Am J Cardiol.

[17] Sakaguchi T, Furukawa T, Shinouchi K, Miura H, Miyazaki K, Hamano G (2013). The efficacy of tolvaptan in acute heart failure with nephrosis. J Card Fail.

[18] Lester SJ, Ryan EW, Schiller NB, Foster E (1999). Best method in clinical practice and in research studies to determine left atrial size. Am J Cardiol.

[19] Tsang TS, Barnes ME, Gersh BJ, Bailey KR, Seward JB (2002). Left atrial volume as a morphophysiologic expression of left ventricular diastolic dysfunction and relation to cardiovascular risk burden. Am J Cardiol.

[20] Segers VF, Brutsaert DL, De Keulenaer GW (2012). Pulmonary hypertension and right heart failure in heart failure with preserved left ventricular ejection fraction: pathophysiology and natural history. Curr Opin Cardiol.

[21] Fukuda S, Saracino G, Matsumura Y, Daimon M, Tran H, Greenberg NL (2006). Three-dimensional geometry of the tricuspid annulus in healthy subjects and in patients with functional tricuspid regurgitation: a real-time, 3-dimensional echocardiographic study. Circulation.

[22] Lorsomradee S, Lorsomradee S, Cromheecke S, ten Broecke PW, De Hert SG (2007). Inferior vena cava diameter and central venous pressure correlation during cardiac surgery. J Cardiothorac Vasc Anesth.

[23] Damman K, Perez AC, Anand IS, Komajda M, McKelvie RS, Zile MR (2014). Worsening renal function and outcome in heart failure patients with preserved ejection fraction and the impact of angiotensin receptor blocker treatment. J Am Coll Cardiol.

[24] Tanaka M, Yoshida H, Furuhashi M, Togashi N, Koyama M, Yamamoto S (2011). Deterioration of renal function by chronic heart failure is associated with congestion and oxidative stress in the tubulointerstitium. Intern Med.

[25] Mullens W, Abrahams Z, Francis GS, Sokos G, Taylor DO, Starling RC (2009). Importance of venous congestion for worsening of renal function in advanced decompensated heart failure. J Am Coll Cardiol.

[26] Maeder MT, Holst DP, Kaye DM (2008). Tricuspid regurgitation contributes to renal dysfunction in patients with heart failure. J Card Fail.

[27] Damman K, Navis G, Smilde TD, Voors AA, van der Bij W, van Veldhuisen DJ (2007). Decreased cardiac output, venous congestion and the association with renal impairment in patients with cardiac dysfunction. Eur J Heart Fail.

[28] Doty JM, Saggi BH, Sugerman HJ, Blocher CR, Pin R, Fakhry I (1999). Effect of increased renal venous pressure on renal function. J Trauma.

[29] Winton FR (1931). The influence of venous pressure on the isolated mammalian kidney. J Physiol.

[30] Shirakabe A, Hata N, Yamamoto M, Kobayashi N, Shinada T, Tomita K (2014). Immediate administration of tolvaptan prevents the exacerbation of acute kidney injury and improves the mid-term prognosis of patients with severely decompensated acute heart failure. Circ J.

[31] Ishikawa M, Kobayashi N, Sugiyama F, Onoda S, Ishimitsu T (2013). Renoprotective effect of vasopressin v2 receptor antagonist tolvaptan in Dahl rats with end-stage heart failure. Int Heart J.

[32] Morooka H, Iwanaga Y, Tamaki Y, Takase T, Akahoshi Y, Nakano Y (2012). Chronic administration of oral vasopressin type 2 receptor antagonist tolvaptan exerts both myocardial and renal protective effects in rats with hypertensive heart failure. Circ Heart Fail.

[33] Onogawa T, Sakamoto Y, Nakamura S, Nakayama S, Fujiki H, Yamamura Y (2011). Effects of tolvaptan on systemic and renal hemodynamic function in dogs with congestive heart failure. Cardiovasc Drugs Ther.

[34] Costello-Boerrigter LC, Boerrigter G, Cataliotti A, Harty GJ, Burnett JC (2010). Renal and anti-aldosterone actions of vasopressin-2 receptor antagonism and B-type natriuretic peptide in experimental heart failure. Circ Heart Fail.

[35] Okada T, Sakaguchi T, Hatamura I, Saji F, Negi S, Otani H (2009). Tolvaptan, a selective oral vasopressin V2 receptor antagonist, ameliorates podocyte injury in puromycin aminonucleoside nephrotic rats. Clin Exp Nephrol.

[36] Eshaghian S, Horwich TB, Fonarow GC (2006). Relation of loop diuretic dose to mortality in advanced heart failure. Am J Cardiol.

[37] Imamura T, Kinugawa K, Shiga T, Kato N, Muraoka H, Minatsuki S (2013). Novel criteria of urine osmolality effectively predict response to tolvaptan in decompensated heart failure patients: Association between non-responder and chronic kidney disease. Circ J.

[38] Iwahashi N, Ebina T, Kimura K (2014). RV dysfunction plays an important role in predicting non-response to tolvaptan in patients with heart failure with reduced ejection fraction. J Card Fail.

[39] Kajimoto K, Abe T (2014). Blood urea nitrogen as a marker of the acute response to addition of tolvaptan to standard therapy in patients hospitalized for acute heart failure syndromes. Int J Cardiol.

